# A case of radiation-induced osteosarcoma treated effectively by boron neutron capture therapy

**DOI:** 10.1186/s13014-014-0237-z

**Published:** 2014-11-04

**Authors:** Gen Futamura, Shinji Kawabata, Hiroyuki Siba, Toshihiko Kuroiwa, Minoru Suzuki, Natsuko Kondo, Koji Ono, Yoshinori Sakurai, Minoru Tanaka, Tomoki Todo, Shin-Ichi Miyatake

**Affiliations:** Department of Neurosurgery, Osaka Medical College, Takatsuki, Japan; Particle Radiation Oncology Research Center, Kyoto University Research Reactor Institute, Kumatori, Japan; Division of Radiation Life Science, Kyoto University Research Reactor Institute, Kumatori, Japan; Division of Innovative Cancer Therapy, and Department of Surgical Neuro-Oncology The Institute of Medical Science, The University of Tokyo, Tokyo, Japan; Cancer Center, Osaka Medical College, Takatsuki, Japan

## Abstract

We treated a 54-year-old Japanese female with a recurrent radiation-induced osteosarcoma arising from left occipital skull, by reactor-based boron neutron capture therapy (BNCT). Her tumor grew rapidly with subcutaneous and epidural extension. She eventually could not walk because of cerebellar ataxia. The tumor was inoperable and radioresistant. BNCT showed a marked initial therapeutic effect: the subcutaneous/epidural tumor reduced without radiation damage of the scalp except hair loss and the patient could walk again only 3 weeks after BNCT. BNCT seems to be a safe and very effective modality in the management of radiation-induced osteosarcomas that are not eligible for operation and other treatment modalities.

## Introduction

The incidence of radiation-induced sarcoma has been estimated to be between 0.03% and 0.3% of all patients who have received radiation therapy [1,2]. Radiation-induced osteosarcomas are being encountered more frequently as the use of radiation therapy becomes more common, and the number of long-term cancer survivors has increased. The original diagnostic criteria for radiation-induced osteosarcomas were proposed in 1948 by Cahan et al. [3], and a short latency period was recently accepted for these tumors [1,4,5]. The diagnosis of radiation-induced osteosarcoma must fulfill the following four criteria: (1) the sarcoma must arise in a previously irradiated field, (2) the sarcoma must be histologically distinct from the original neoplasm, (3) there was no evidence of tumor in the involved bone at the time of initial irradiation, and (4) there must be a latency period between the irradiation and the development of the sarcoma at least 3 years.

Radiation-induced osteosarcoma of the head is a devastating complication of radiation therapy. It is very rare but aggressive, with high recurrence and a poor prognosis [6]. The median overall survival time was reported to be 29 months [1]. Osteosarcoma is thought to be radioresistant [7,8]. Therefore, complete surgical resection has been described as the most important prognostic factor [9] and the first choice of treatment for radiation-induced osteosarcoma. However, if complete surgical resection is difficult (as it was in the present case), adjuvant chemotherapy and radiotherapy should be considered. These therapeutic effects have thus far been found to be insufficient, however. We report here the case of a patient with recurrent radiation-induced osteosarcoma who was treated effectively by boron neutron capture therapy (BNCT).

BNCT is based on the nuclear capture reactions that occur when non-radioactive boron-10 is irradiated with neutrons of the appropriate energy to yield high linear energy transfer (LET) alpha particles (4He) and recoiling lithium-7 (7Li) nuclei. Since these particles have short path-lengths of approximately one cell diameter, their lethality is primarily limited to boron-containing cells. Theoretically, high LET particles have the advantage to overcome radioresistance to photon radiotherapies (such as X-rays). BNCT can thus be regarded as tumor cell-selective and an intensive particle radiation modality with minimal damage to normal tissue, [10,11] even for X-ray-resistant tumors. Here we report a successfully treated a case of radiation-induced osteosarcoma by reactor-based BNCT.

## Case report

**Figure 1:**
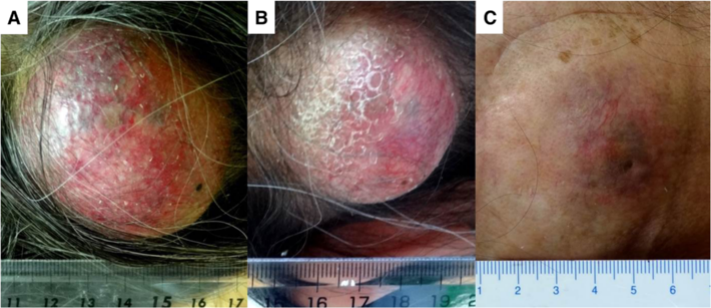
**Marked improvement of the subcutaneous tumor at 3 weeks after the application of BNCT. A**: Just prior to the BNCT; the tumor is elastic hard, and painful. **B**: Seven days after the BNCT; the tumor is soft and no longer painful. **C**: At 2 months after the BNCT, the tumor had shrunk drastically without radiation damage to the skin.

**Figure 2:**
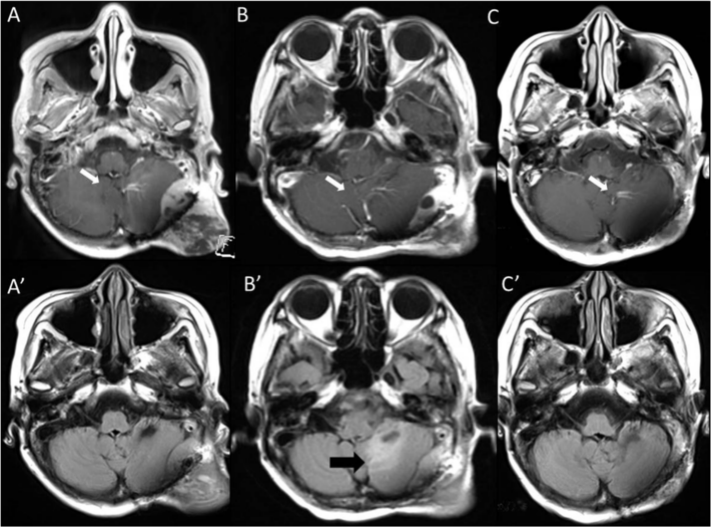
**MRI of the patient’s brain before and after the BNCT.** White arrows indicate a venous angioma, which was recognized incidentally and judged as a sectional standard of MRI. **A**: Gd-enhanced T1-weighted MRI of the brain 1 month before the BNCT. There was a subcutaneous and epidural tumor mass. **B**: Gd-enhanced T1-weighted MRI at 4 days after BNCT. The tumor mass was reduced. **C**: Gd-enhanced T1-weighted MRI of the brain 3 months after BNCT. The tumor mass was further reduced. **A**’: Fluid-attenuated inversion recovery (FLAIR) MRI of the brain 1 month before BNCT. **B**’: FLAIR MRI of the brain 4 days after BNCT. The tumor mass was reduced, but the edema had worsened. A black arrow indicates the cerebellar edema. **C**’: FLAIR MRI of the brain 3 months after BNCT. The tumor mass was further reduced, and the edema had disappeared.

A 54-year-old Japanese female was referred to our institute for treatment by BNCT of a recurrent radiation-induced osteosarcoma involving the left occipital bone. Ten years earlier, she was diagnosed with cancer of the uterine body and underwent resection surgery. Two years after that surgery, she underwent chemotherapy and whole-brain radiation therapy (WBRT, total 30 Gy with 10 fractions) including the cerebellum for brain metastasis. Six years after the WBRT, she was diagnosed with a radiation-induced osteosarcoma involving the left occipital bone, and she underwent resection surgery and successive chemotherapy using methotrexate. One year after that surgery and chemotherapy, the subcutaneous tumor appeared again in the left occipital region and rapidly enlarged over a period of only 3 months (Figure 1A). Magnetic resonance images (MRI) showed the epidural tumor invasion (Figure 2A and A’). Eventually, the patient could not walk because of acutely developing cerebellar ataxia. This tumor was diagnosed as a recurrence of the radiation-induced osteosarcoma in accord with the above Cahan’s criteria [3].

**Figure 3:**
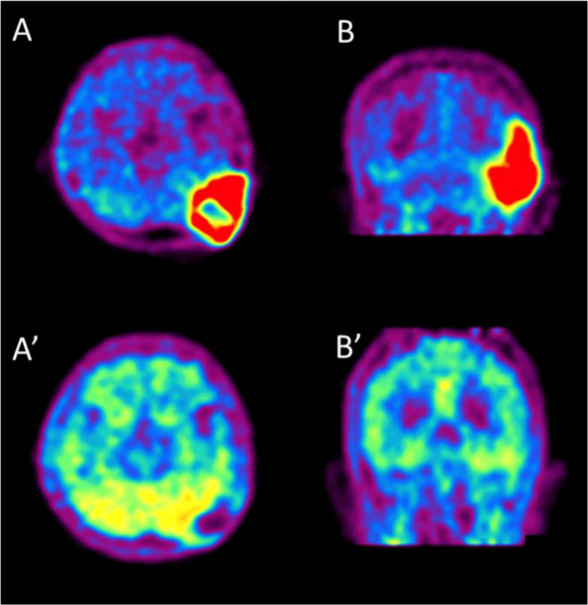
**Fluoride-labeled boronophenylalanine-PET imaging of the brain before and after BNCT.** Fluoride-labeled boronophenylalanine-PET imaging taken 1 month prior to BNCT (**A** and **B**) and 2 months after BNCT (**A**’ and **B**’). **A** and **A**’: axial imaging, **B** and **B**’: coronal imaging. In **A** and **B**, L/N ratio was calculated as 5.0. This is theoretical proof of tumor selective destruction using BPA in BNCT. Also absorbed doses were simulated with this L/N ratio. 2 months after BNCT, **A**’ and **B**’ show the decreased L/N ratio as 1.2, indicating the marked effectiveness.

We performed BNCT for the radiation-induced osteosarcoma because the lesion/normal brain (L/N) ratio of fluoride-labeled boronophenylalanine positron emission tomography (FBPA-PET) was enough high, as shown in Figure 3A and B (L/N ratio: 3.8) [12]. For the BNCT, neutron irradiation was applied at Kyoto University Reactor.

The patient was administered 500 mg/kg of BPA intravenously for 3.2 hours (200 mg/kg for initial 2 hours, prior to neutron irradiation, 100 mg/kg for 1.2 hours during neutron irradiation). The boron concentration in the blood was monitored by sampling every 1 hour after boron compound administration until neutron irradiation was completed. The boron concentrations from BPA in the tumor and normal brain were estimated from the L/N ratio of 18 F-BPA on PET. The neutron fluence rate was simulated by the dose-planning system, SERA (Idaho National Engineering and Environmental Laboratory, Idaho Falls, ID) and the total doses to the tumor and normal brain were simulated. The neutron irradiation time was determined not to exceed 13 Gy-Eq to the normal brain in accordance with our recent protocol of BNCT for high-grade meningiomas [10]. For this case, irradiation time was 70 minutes and B10 concentration of the venous blood was judged as 37.2 ppm during the neutron irradiation. Here, Gy-Eq (Gy: Gray) means an X-ray dose that can give biologically equivalent effects to total BNCT radiation. The scalp just above the tumor was covered with the bolus composed of sodium polyacrylate with 1 cm-thickness to gain the superficial neutron flux. After the treatment, the doses given were re-estimated precisely and are shown in Table 1. We hypothesized the boron concentrations of the blood, brain, and skin were equal, as we did in the previous BNCT. RBE and CBE values employed here were listed in Table 2.

**Table 1:** **Estimated dose distribution at the central axis of neutron-irradiation field**

**Depth (cm)**	**Total dose (tumor) (Gy-eq)**	**Total dose (skin) (Gy-eq)**	**Total dose (mucosa) (Gy-eq)**	**Total dose (brain) (Gy-eq)**	**Thermal neutron (Gy-eq)**	**Fast neutron (Gy-eq)**	**γ-ray (Gy-eq)**	**Boron dose (tumor) (Gy-eq)**
0.00	5.28E + 01	1.24E + 01	2.08E + 01	8.37E + 00	5.05E-01	2.13E + 00	1.00E + 00	4.92E + 01
0.50	6.79E + 01	----------	2.61E + 01	9.90E + 00	6.56E-01	1.87E + 00	1.22E + 00	6.41E + 01
1.00	8.06E + 01	----------	3.06E + 01	1.12E + 01	7.83E-01	1.64E + 00	1.43E + 00	7.67E + 01
1.50	8.47E + 01	----------	3.20E + 01	1.16E + 01	8.24E-01	1.35E + 00	1.63E + 00	8.09E + 01
2.00	9.00E + 01	----------	3.39E + 01	1.21E + 01	8.77E-01	1.17E + 00	1.80E + 00	8.62E + 01
2.50	9.38E + 01	----------	3.53E + 01	1.26E + 01	9.13E-01	1.11E + 00	1.92E + 00	8.98E + 01
3.00	9.55E + 01	----------	3.58E + 01	1.27E + 01	9.31E-01	9.77E-01	2.02E + 00	9.16E + 01
3.50	9.53E + 01	----------	3.57E + 01	1.27E + 01	9.30E-01	8.63E-01	2.09E + 00	9.14E + 01
4.00	9.18E + 01	----------	3.44E + 01	1.22E + 01	8.94E-01	7.72E-01	2.11E + 00	8.80E + 01
4.50	8.62E + 01	----------	3.24E + 01	1.16E + 01	8.38E-01	6.91E-01	2.10E + 00	8.26E + 01
5.00	7.97E + 01	----------	3.00E + 01	1.08E + 01	7.74E-01	6.18E-01	2.08E + 00	7.62E + 01
5.50	7.15E + 01	----------	2.70E + 01	9.79E + 00	6.93E-01	5.54E-01	1.99E + 00	6.82E + 01
5.80	6.77E + 01	----------	2.56E + 01	9.31E + 00	6.55E-01	5.12E-01	1.95E + 00	6.45E + 01

**Table 2:** **RBE (relative biological effectiveness) factor**

**Radiation**	**Tumor**	**Brain**	**Skin**
Thermal neuton	3.0	3.0	3.0
Epithermal neutron	3.0	3.0	3.0
^10^B (n,α)^7^ Li: BPA	3.8	1.35	2.5
γ-ray dose	1.0	1.0	1.0

Absorbed physical dose and X-ray-equivalent dose (Gy-Eq) are calculated with the following formula;

$$ {\mathrm{E}}_{\mathrm{Total}}={\mathrm{E}}_{\mathrm{B}10}+{\mathrm{E}}_{\mathrm{Thermal}}+{\mathrm{E}}_{\mathrm{Fast}}+{\mathrm{E}}_{\upgamma} $$

$$ {\mathrm{E}}_{\mathrm{B}10}=\left({\mathrm{C}}_{\mathrm{B}\mathrm{SH}}\times \mathrm{C}\mathrm{B}{\mathrm{E}}_{\mathrm{B}\mathrm{SH}}+{\mathrm{C}}_{\mathrm{B}\mathrm{PA}}\times \mathrm{C}\mathrm{B}{\mathrm{E}}_{\mathrm{B}\mathrm{PA}}\right)\times 7.43\times {10}^{-14}\times {\Phi}_{\mathrm{Thermal}} $$

$$ {\mathrm{E}}_{\mathrm{Thermal}}=\mathrm{N}\times \mathrm{R}\mathrm{B}{\mathrm{E}}_{\mathrm{Thermal}}\times 6.78\times {10}^{-14}\times {\Phi}_{\mathrm{Thermal}} $$

$$ {\mathrm{E}}_{\mathrm{Fast}}=\mathrm{R}\mathrm{B}{\mathrm{E}}_{\mathrm{Fast}}\times {\mathrm{D}}_{\mathrm{Fast}} $$

$$ {\mathrm{E}}_{\upgamma}=\mathrm{R}\mathrm{B}{\mathrm{E}}_{\upgamma}\times {\mathrm{D}}_{\upgamma} $$

D: physical absorbed dose (Gy),

ΦThermal: fluence of theraml neutron (cm-2),

N: nitrogen concentration (2%, here)

C: B10 concentration (ppm).

For this patient, we estimated that the minimum tumor and maximum normal brain and skin doses were 67.7, 12.7 and 12.4 Gy-Eq, respectively in the BNCT, simulated from F-BPA-PET imaging and the blood BPA concentration (Table 1).

At one day after the BNCT, the patient’s gait disturbance was aggravated. Computed tomography at that time showed aggravation of peri-lesional edema (data not shown). Remarkably, the MRI taken 4 days after the BNCT demonstrated the definitive shrinkage of the mass, but the left cerebellar edema was still there (Figure 2B and B’). We then treated the edema with dehydrators and steroids. The symptoms gradually improved.

At only 3 weeks after the BNCT, the patient was able to walk again stably without aid. The subcutaneous tumor was reduced dramatically without radiation injury of the scalp, with time after BNCT, as shown in Figure 1B and C. The only adverse effect was hair loss in neutron-irradiation field, as shown in Figure 1C. MRI showed the further reduction of tumor and the disappearance of the cerebellar edema (Figure 2C and C’), 3 months after BNCT. Also F-BPA-PET taken 2 months after BNCT showed faint tracer uptake, indicating some metabolic change at least by this treatment (Figure 3A’ and B’, L/N ratio as 1.2).

## Discussion

Radiation-induced osteosarcoma is not common. It has an aggressive nature, high recurrence rate, and poor prognosis. A standard therapy protocol has not yet been established for non-resectable tumors, but it was reported that particle radiotherapy (treatment with proton and carbon beams) had a therapeutic effect on these tumors [7,13].

In the present case, the tumor was chemo-resistant and difficult to totally resect because it invaded the left transverse and sigmoid venous sinuses. In addition, the subcutaneously extended tumor invaded the surface of the skin, and we thus suspected that a skin deficit due to surgery was inevitable and that particle radiotherapy for this tumor was likely to cause severe radiation-induced adverse effects on the scalp. The tumor was radiation-induced, and the cerebellum and overlying scalp had a history of X-ray treatment. Moreover, osteosarcomas have the characteristic of being radioresistant, i.e., X-ray-resistant. In light of these medical circumstances, we chose BNCT as the treatment modality for this patient. In the present case, the patient was successfully treated by BNCT without skin damage even though her tumor invaded the superficial scalp.

We recently reported the effectiveness of BNCT for radiation-refractory high-grade meningiomas [10]. In that report, we speculated that the difference in tumor shrinkage between the alpha and lithium particles provided by BNCT and other particles such as carbon and protons may be ascribed to the difference in LET noted above and their fraction size [10].

Other types of particle radiotherapy and some stereotactic radiotherapies which have been tried recently for tumors were applied as multi-fraction. The reduction of the tumor mass was thus not very prominent, and it was difficult to improve the patients’ symptoms by means other than BNCT. BNCT can deliver high dose particles in a tumor-selective fashion in a single session, and in some cases the resulting reduction of the tumor was fast; this rapid shrinkage might contribute to the prompt elimination of symptoms [10]. Indeed, the present patient, within a very short time, exhibited improvement of her gait disturbance due to cerebellar ataxia.

Only a couple of articles were published with regard to pre-clinical study of BNCT for osteosarcoma in in vitro cell culture and animal experiments [14-17]. Among them, Russian research group reported successful treatment of dog osteosarcoma case by BNCT. Also only one preliminary report was published with regard to a BNCT-treated osteosarcoma case in head and neck region with limited description, so far [18]. We are not sure of the compound biological effectiveness (CBE) of BPA for osteosarcomas, and we were only able to estimate CBE as being the same for glioblastoma (i.e., 3.8) [19] as we did for high-grade meningioma [10]. For the estimation of the prescribed dose for this case, we adopted the reported value of CBE and relative biological effectiveness of neutron itself for tumors and normal tissues [20]. Thereafter the estimated tumor dose was uncertain in this case. However, as a result of the BNCT, the tumor shrank rapidly, the patient’s clinical symptoms improved, metabolically scarce uptake of the amino-acid tracer was observed in the follow-up PET imaging and no serious damage was observed in the scalp and brain, so far at 6 months after BNCT, although the observation period was short.

Based on this outcome, we found that BNCT was an effective treatment for our patient. However, careful follow-up or the use of bevacizumab may be necessary in some cases [21], because WBRT that has been already performed may cause brain radiation necrosis.

We experienced only a case of successful treatment of BNCT for radiation-induced osteosacoma. Hopefully these potential therapeutic effects will be applicable for non-radiation-induced osteosarcomas which are generally refractory for other treatment modalities.

## Conclusions

BNCT is an effective treatment for non-resectable radiation-induced skull osteosarcoma. We suggest that BNCT is the only effective therapy for tumors that have invaded the skin. Further applications of BNCT for similar cases are expected.
